# In-Home Mobility Frequency and Stability in Older Adults Living Alone With or Without MCI: Introduction of New Metrics

**DOI:** 10.3389/fdgth.2021.764510

**Published:** 2021-10-26

**Authors:** Chao-Yi Wu, Hiroko H. Dodge, Christina Reynolds, Lisa L. Barnes, Lisa C. Silbert, Miranda M. Lim, Nora Mattek, Sarah Gothard, Jeffrey A. Kaye, Zachary Beattie

**Affiliations:** ^1^Department of Neurology, Oregon Health & Science University, Portland, OR, United States; ^2^Oregon Center for Aging & Technology (ORCATECH), Oregon Health & Science University, Portland, OR, United States; ^3^Department of Neurological Sciences, Rush Medical College, Chicago, IL, United States; ^4^Rush Alzheimer's Disease Center, Rush Medical College, Chicago, IL, United States; ^5^Department of Neurology, Veterans Affairs Portland Health Care System, Portland, OR, United States; ^6^Department of Behavioral Neuroscience, Oregon Health & Science University, Portland, OR, United States; ^7^Department of Medicine, Oregon Health & Science University, Portland, OR, United States; ^8^Oregon Institute of Occupational Health Sciences, Oregon Health & Science University, Portland, OR, United States; ^9^National Center for Rehabilitative Auditory Research, Veterans Affairs Portland Health Care System, Portland, OR, United States

**Keywords:** gait speed, indoor mobility, passive monitoring, sensing technologies, life space, Alzheimer's disease, movement

## Abstract

**Background:** Older adults spend a considerable amount of time inside their residences; however, most research investigates out-of-home mobility and its health correlates. We measured indoor mobility using room-to-room transitions, tested their psychometric properties, and correlated indoor mobility with cognitive and functional status.

**Materials and Methods:** Community-dwelling older adults living alone (*n* = 139; age = 78.1 ± 8.6 years) from the Oregon Center for Aging & Technology (ORCATECH) and Minority Aging Research Study (MARS) were included in the study. Two indoor mobility features were developed using non-parametric parameters (frequency; stability): Indoor mobility frequency (room-to-room transitions/day) was detected using passive infrared (PIR) motion sensors fixed on the walls in four geographic locations (bathroom; bedroom; kitchen; living room) and using door contact sensors attached to the egress door in the entrance. Indoor mobility stability was estimated by variances of number of room-to-room transitions over a week. Test-retest reliability (Intra-class coefficient, ICC) and the minimal clinically important difference (MCID) defined as the standard error of measurement (SEM) were generated. Generalized estimating equations models related mobility features with mild cognitive impairment (MCI) and functional status (gait speed).

**Results:** An average of 206 days (±127) of sensor data were analyzed per individual. Indoor mobility frequency and stability showed good to excellent test-retest reliability (ICCs = 0.91[0.88–0.94]; 0.59[0.48–0.70]). The MCIDs of mobility frequency and mobility stability were 18 and 0.09, respectively. On average, a higher indoor mobility frequency was associated with faster gait speed (β = 0.53, *p* = 0.04), suggesting an increase of 5.3 room-to-room transitions per day was associated with an increase of 10 cm/s gait speed. A decrease in mobility stability was associated with MCI (β = −0.04, *p* = 0.03).

**Discussion:** Mobility frequency and stability in the home are clinically meaningful and reliable features. Pervasive-sensing systems deployed in homes can objectively reveal cognitive and functional status in older adults who live alone.

## Introduction

Mobility, the ability to move about in one's environment, is fundamental to sustaining independence and well-being. The notion of human mobility is often measured through the geographic extent of movements beyond the home space (1–3). As one ages, the proportion of time spent beyond the home becomes more susceptible to cognitive and functional changes (4–9). It is estimated that community-dwelling older adults spend approximately 83% of their time at home (10, 11), compared to 61–77% of the time spent at home by middle-aged adults (12). Using motion-activity sensors, a study of primarily octogenarian healthy older adults documents an average of 20.5 h spent in-home per day (13). Although older adults spend a considerable amount of time inside their residence, most research investigates out-of-home mobility and its health correlates. There are fundamental differences between indoor and out-of-home mobility. Individuals carry out different activities of daily living (ADL) when moving around indoors (bathing, preparing meals, using a computer) vs. time spent out of the home (shopping, visiting friends, driving). While out-of-home mobility may reveal the ability to carry out higher-order cognitive tasks, indoor mobility might delineate everyday cognition/function to support a combination of basic and more complex needs. Assessing indoor mobility and identifying its cognitive and functional correlates may aid in early detection of health changes (9, 14), especially for those with impairment who either live alone or do not have care partners.

In prior work, room-level activity distributions are observed to differentiate older adults with mild cognitive impairment (MCI) from those without cognitive impairment. One study found that indoor movement measured by the number of motion sensor activations decreased along with cognitive decline over a year of monitoring (15). Other studies used the probability of a person being in specific rooms of the home to predict the transition to MCI (16, 17). The authors were able to detect MCI with an average area under the curve of 0.72 using activity models estimated over 12 weeks. A study conducted in a nursing home found that cognitive status was associated with the number of times older adults move around inside the nursing home (18). Another study also found that those with dementia exhibited unorganized indoor behavior patterns as compared to those with intact cognition over 20 days of monitoring (19). Although these studies have collected indoor mobility data, their analyses and feature extraction often overlooked the stability and variability aspects of indoor mobility over the course of the day and weeks.

Here we investigate the metric of indoor mobility using “room-to-room transitions” (20). Room-to-room transition comprises two aspects: (1) indoor geographic locations and (2) transitions within a residence (18, 21). The indoor geographic locations include commonly used areas in the home such as bathrooms, bedrooms, kitchen, living room, and entrance. “Transitions” describes a person navigating between these indoor areas, within each hour, throughout the day. Therefore, indoor mobility can be derived by the number of room transitions over the course of a day (“mobility frequency”) and other measures such as the variances of the number of room transitions across several days (“mobility stability”). Since mobility patterns of older individuals in their home show a high degree of predictability and regularity (22), discontinuities in established room-level mobility patterns may provide an opportunity to predict individual human health and functional status or detect adverse events and trends. We thus hypothesize that indoor mobility stability might characterize a person's cognitive status to regulate their rhythms of everyday activities, and changes in the stability of this measure reflect transitions to MCI.

To test this hypothesis, we assess hourly indoor mobility using room-to-room transitions to understand the association between in-home mobility and aspects of health in older adults who live alone. Using an unobtrusive in-home sensing platform developed by the Oregon Center for Aging and Technology (ORCATECH) (23, 24), we remotely detect the navigation of indoor geographic (room) locations of a person. The ubiquitous and continuous sensing platform allows monitoring behaviors without adding burden on participants and collecting longitudinal high-resolution room-level data. The methods and algorithms for estimating mobility frequency and mobility stability in personal residences are described in this manuscript. To ensure these mobility features have sufficient psychometric properties, we test their test-retest reliability and the minimal clinically important difference (MCID). We then compare whether older adults with various cognitive (mild cognitive impairment, MCI) and functional statuses (gait speed) exhibit different indoor mobility frequency and mobility stability to confirm external validity.

## Materials and Methods

### Participants

Participants were recruited from two longitudinal aging cohort studies, one at Oregon Health & Science University and one at Rush University. At Oregon Health & Science University, ORCATECH has led longitudinal studies examining the use of unobtrusive in-home sensing technology to detect early cognitive decline in community-dwelling older adults (23–27). At Rush University, the Minority Aging Research Study (MARS) is a longitudinal cohort study of decline in cognitive function in older African Americans (28). Study approval was obtained from the Institutional Review Boards of Oregon Health & Science University (IRB #2765, #17123, #4089) and Rush University (IRB # L03030302). All participants provided written informed consent.

### Participant Inclusion/Exclusion Criteria

In order to be eligible for the ORCATECH cohort, participants needed to be over 57 years old, living in a residence larger than a one-room apartment, living either alone or with a spouse/partner, and without dementia [age and education adjusted Montreal Cognitive Assessment MoCA > 18 (29) and Clinical Dementia Rating, CDR score 0 or 0.5 (30)]. To be eligible for the MARS study, participants had to self-report race as African American, be 65 years of age or older, and be without a dementia diagnosis (31) or taking dementia medications. All the households needed to have a reliable broadband internet connection. Study exclusion criteria for both cohorts included conditions that would limit their physical participation (e.g., wheelchair-bound). In the current study, only ORCATECH or MARS participants who lived alone were included. One hundred and forty three participants met inclusion criteria and were included in the current study. Excluding those with missing clinical data (*n* = 4), a total of 139 homes/participants were included in the analysis.

### Mobility Frequency and Mobility Stability Inside the Home

In-home passive infrared (PIR) motion sensors were fixed on the wall in four major rooms [bathroom(s); bedroom(s); kitchen(s); living room(s)]. Contact sensors were also fixed on the front door (entrance). The in-home sensor platform and protocol for data collection have previously been detailed (23, 24). Each room and door were assigned unique identifiers. The firings of sensors in rooms and on the front door were independent. Therefore, we were able to estimate the presence of a participant within a specific room or entrance. The algorithms of indoor mobility frequency (number of room-to-room transitions) were described elsewhere (22). In brief, we first identified all the in-room and front door firings per day. If a different room or door firing followed a room firing, then the time of the firing was marked as one transition. For example, if the series of transitions was: bedroom → kitchen → entrance → living room, the number of transitions would be 3. To ensure the movement was a purposeful transition rather than passing by or a random walk, transitions in and out of a room within 20 seconds of each other were excluded. For example, if the series of transitions was: kitchen → entrance → living room and the time between the first transition (kitchen → entrance) and second transition (entrance → living room) was ≤ 20 seconds, then the number of transitions would be 1 (kitchen → living room) and not 2 as it is likely the participant was just passing through the entrance. This decision was guided based on a previous study using a cutoff of 30 seconds to define a meaningful transition for nursing home residents (18). Since our sample was composed of community-dwelling relatively healthy older adults, we used 20 seconds as a cutoff of a meaningful transition. With this data, we then estimated the total number of room-to-room transitions over a day as the indoor mobility frequency.

Indoor mobility stability was quantified by calculating the variance of number of room-to-room transitions within a given week. This approach has been used in circadian rhythm research to quantify the stability of the sleep-wake cycle (32). We adopted the algorithm since we also measured 24-h mobility frequency at home. The interdaily indoor mobility stability was computed as a non-parametric variable for subject *i*:


$$\begin{array}{l} Interdaily\;indoor\;movement\;stability=\;\frac{n\sum_{h\;=\;1}^{p}{\left(\bar{x_{ih}}-\bar{x_{i}}\right)}^{2}}{p\sum_{k\;=\;1}^{n}{\left(x_{ik}-\bar{x_{i}}\right)}^{2}} \end{array}$$

Where *n* is the total number of hourly data points per week (7^*^24 = 168 in this case), *p* is the number of hourly data points per day (24 in this case), $\bar{x_{ih}}$ are the hourly means for the specific subject, $\bar{x_{i}}$ is the mean of all data within a week for the specific subject, and *x*_*i*_ are the individual data points.

In the study, participants completed a health questionnaire that was automatically emailed to them once weekly. The questionnaire collected health and life event information such as the severity of pain, low mood (feeling “blue”), whether they had any overnight visitors during the past week, or whether they were away from home overnight. We extracted all the weekly online surveys answered by participants. With the date of completed surveys, we were able to match weekly survey data with daily sensor data. In order to only include observations from the study participants, data were excluded from days when overnight visitors were reported, or they were away from home overnight. Data collected after the Coronavirus Disease 2019 (COVID-19 pandemic) (the date of governor's proclaiming stay at home orders: March 23, 2020 in Oregon) were also excluded since COVID-19 related restrictions may impact the data and participants' daily routines.

### Mild Cognitive Impairment (MCI)

For the ORCATECH cohort, MCI was defined by the CDR score of 0.5 at an annual assessment. For the MARS study, MCI was determined using a two-stage process by an experienced clinician. First, a computer algorithm was used to rate impairment in five cognitive domains (episodic memory, semantic memory, working memory, perceptual speed, and visuospatial ability). Second, after reviewing cognitive data, occupation, years of education, and motor and sensory problems, a neuropsychologist made the final decision, as previously described (31).

### Gait Speed

For the ORCATECH cohort, in-person observed gait speed was measured by a timed 4.6 meter (15 foot) out and back gait test at a usual pace during an annual assessment. The total time (s) to complete the usual paced walk was recorded, with less time indicating a faster gait speed. For the MARS study, gait speed was based on the time to walk 2.4 meters (8 foot) assessed at each annual assessment; the gait speed closest in time to the extraction of mobility metrics was used for the current analysis. Gait speed measures from the two studies were harmonized using the same unit (cm/sec).

### Covariates

#### Demographic Variables

Participant characteristics (age, gender, race, years of education) were collected at baseline. The number of rooms in the house was estimated using the number of PIR motion sensors installed in the home as the ORCATECH sensor deployment protocol dictates installing one PIR motion sensor per room.

#### Weekly Health and Contextual Variables

Weekly health (pain interfering with daily activities, low mood) was collected from the self-reported weekly online surveys. These variables were collected because they might impact our indoor mobility outcome measures and were therefore used as covariates in the subsequent analyses where indicated. For the pain interference question, participants were asked, “During the past week, how much did pain interfere with your normal activities or work (including both work outside the home and housework)?” Pain interference scores ranged from 0 to 5, with 0 being not at all to 5 being severely interfering with daily lives. Low mood (“Blueness”) was identified via a self-reported question: “Have you felt downhearted or blue for three or more days in the past week? (Yes/No).”

Two contextual environmental variables were collected because they had the potential to impact our indoor mobility outcome measures. The time spent out-of-home was calculated by the time difference between two door openings when no one was home. A more in-depth description of the algorithm used to extract out-of-home activities (i.e., when no one was home) from the PIR motion sensors was described previously (10). The hours of daylight were calculated based on the latitude of residence and the week of the year. These contextual environmental variables are also controlled in the analyses.

### Statistical Analysis

Characteristics at baseline (first week of data) were compared between MCI and non-MCI participants using the Student *t* test and the Pearson χ2 test for categorical variables. Effect size Cohen's *d* was calculated to estimate the magnitude of baseline difference in indoor mobility frequency and mobility stability between MCI and non-MCI groups.

### Psychometric Properties

The first two weeks of sensor data for each participant were used to examine the test-retest reliability and the minimal clinically important difference (MCID). Test-retest reliability was computed using the intra-class correlation coefficient (ICC) (33). ICCs are often interpreted as follows: poor (ICC < 0.4), fair to good (0.4 ≤ ICC < 0.75), and excellent (ICC ≥ 0.75) (34). The MCID, or absolute reliability, was established using the standard error of measurements (SEM) (35). The SEM was computed as follows (36): SEM = SD_baseline_ × √ (1 – ICC). For future studies, the absolute differences between treatment groups in mobility frequency and mobility stability should be larger than MCID values to confirm treatment efficacy rather than measurement errors.

### Relationships Between Indoor Mobility and Cognitive and Functional Status

We examined whether gait speed was associated with indoor mobility frequency when controlling for demographics (age, gender, education, race, number of rooms in the house), health (low mood, pain), and contextual factors (time spent out-of-home, hours of daylight). We examined whether indoor mobility stability was different between older adults with and without MCI. Generalized estimating equations (GEE) models were used to explore these two questions. In the GEE model, all repeated weekly observations from each individual were combined, while within-individual correlations were taken into account in estimating standard errors. The dependent outcomes were indoor mobility frequency and mobility stability. Independent variables were gait speed measured in cm/sec and MCI status indicated by a dummy variable (0/1). SAS procedure PROC GEE was used for the analysis.

## Results

A total of 139 participants with 4,964 weeks (30,608 days) of data were analyzed. On average, 35.9 weeks of survey data and 205.9 days of sensor data were analyzed per individual. Participant characteristics are presented in Table 1. Among 139 participants, 19 participants met the criteria for MCI. The MCI group was younger, had a higher proportion of males, and had fewer rooms in their residences than the non-MCI group (Table 1).

**Table 1:** Participant baseline characteristics (*n* = 139).

**Characteristics [n (%)]**	**All**	**Non-MCI**	**MCI**	***t*-statistics/χ^2^-statistics**	***p*-value**
	**139 (100)**	**120 (86.3)**	**19 (13.7)**		
Age [mean (SD)]	78.1 (8.6)	78.9 (8.5)	73.1 (7.5)	*t_(137)_* = 2.83	0.01
Gender (female) [n (%)]	103 (74.1)	94 (78.3)	9 (47.4)	χ^2^*_(1)_* = 8.20	<0.01
Race (White) [n (%)]	107 (77.0)	90 (75.0)	17 (89.5)	χ^2^*_(1)_* = 1.94	0.16
Years of education [mean (SD)]	15.7 (2.8)	15.8 (2.7)	14.9 (3.4)	*t_(137)_* = 1.34	0.18
Gait speed (cm/sec) [mean (SD)]	70.8 (20.5)	70.1 (20.3)	75.6 (22.2)	*t_(137)_* = −1.08	0.28
Number of rooms in the home [mean (SD)]	6.1 (2.2)	6.3 (2.2)	5.0 (1.6)	*t_(137)_* = 2.38	0.02
Data points [mean (SD)]
Number of weekly surveys	35.9 (19.5)	36.2 (19.2)	34.4 (21.9)	*t_(137)_* = 0.36	0.72
Number of days of sensor data	205.9 (127.1)	206.7 (125.9)	200.7 (138.1)	*t_(137)_* = 0.19	0.85
Baseline indoor mobility (first week) [mean (SD)]
Indoor mobility frequency (transitions/day)	112.6 (61.7)	111.1 (63.1)	118.7 (53.6)	*t_(137)_* = −0.50	0.62
Indoor mobility stability	0.4 (0.2)	0.4 (0.1)	0.3 (0.1)	*t_(137)_* = 3.32	<0.01

*MCI (mild cognitive impairment)*.

**Figure 1:**
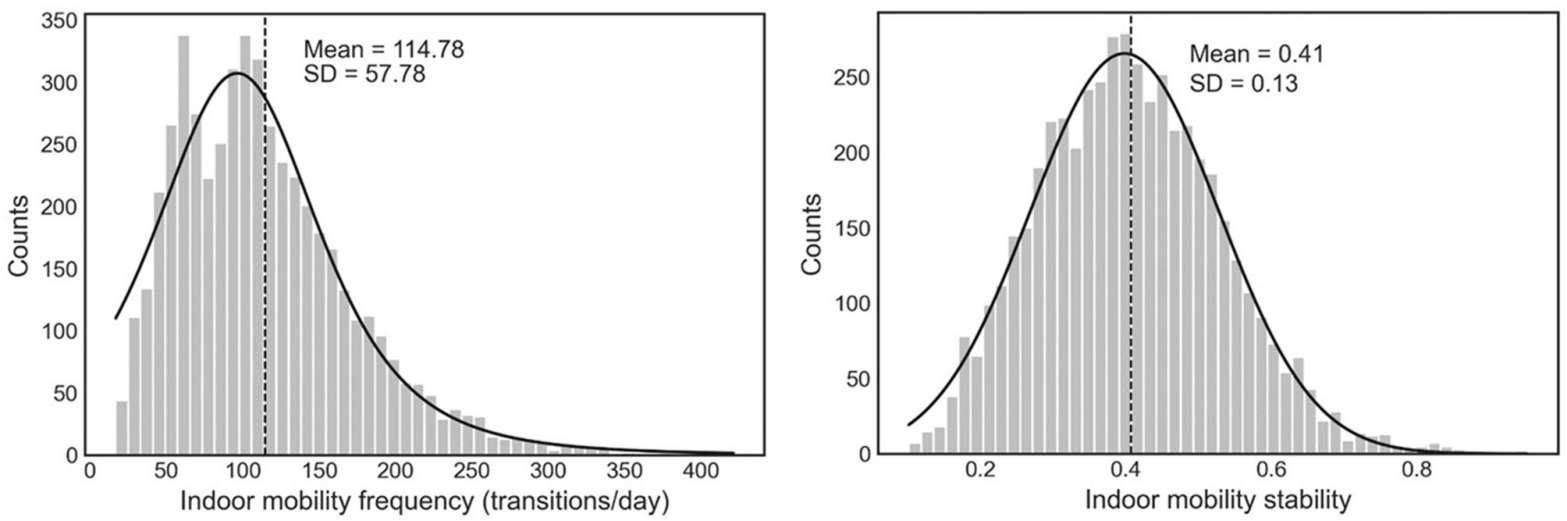
The distribution of indoor mobility frequency and indoor mobility stability (*n* = 4,964 weeks).

Indoor mobility frequency data showed a positively skewed distribution (Figure 1). The daily average mobility frequency was 116.2 ± 61.7 transitions at baseline (first week). There was no significant difference in the indoor mobility frequency between MCI and non-MCI groups (Cohen's *d* = 0.13, t = −0.5, *p* = 0.62, Table 1). Indoor mobility stability data showed a normal distribution (Figure 1). Average mobility stability was 0.4 ± 0.2 at baseline (first week). There was a significant difference in the indoor mobility stability between MCI and non-MCI groups (Cohen's *d* = 1, *t* = 3.32, *p* < 0.01, Table 1), suggesting that 84% of the MCI individuals scored below the mean of the non-MCI group.

### Test-Retest Reliability and the MCID

Indoor mobility frequency showed excellent test-retest reliability, with an ICC [95% Confidence Interval] of 0.91 [0.88–0.94]. The MCID of mobility frequency was 18 (SEM).

Indoor mobility stability showed fair to good test-retest reliability, with an ICC [95% Confidence Interval] of 0.59 [0.48–0.70]. The MCID of mobility stability was 0.09 (SEM).

### GEE Model With the Outcome Being Mobility Frequency

**Figure 2:**
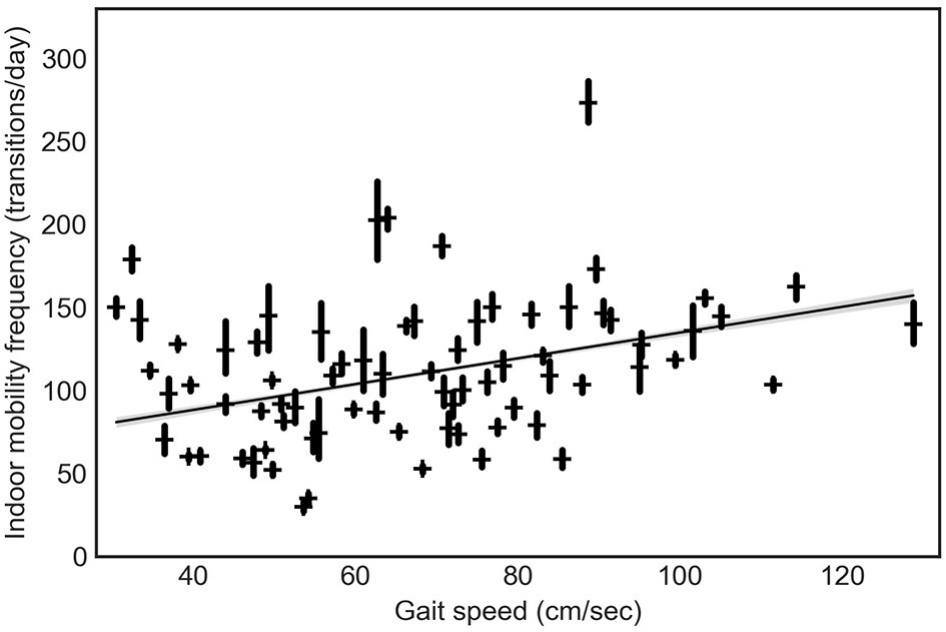
The relationship between indoor mobility frequency and gait speed.

A GEE model revealed that a higher indoor mobility frequency was associated with faster gait speed (β = 0.63, *p* < 0.01) (Figure 2). Results remained statistically significant after adjusting for age, gender, race, education, pain, low mood, number of rooms, hours of daylight, and time out-of-home (β = 0.53, *p* = 0.04), suggesting that an increase of 10 cm/sec of gait speed was associated with an increase of 5.3 room-to-room transitions per day at home (Table 2).

**Table 2:** Generalized estimating equations models with outcomes being indoor mobility frequency and indoor mobility stability (*n* = 4,964 weeks).

**Variable**	**Outcome: Indoor mobility frequency**						**Outcome: Indoor mobility stability**					
	**Model without covariates**			**Model with covariates**			**Model without covariates**			**Model with covariates**		
	**Beta**	**SE**	***p***	**Beta**	**SE**	***p***	**Beta**	**SE**	***p***	**Beta**	**SE**	***p***
Intercept	68.76	14.13	<0.01	225.87	58.01	<0.01	0.42	0.01	<0.01	0.15	0.08	0.06
Gait speed	0.63	0.19	<0.01	0.53	0.26	0.04						
Mild cognitive impairment (MCI)							−0.05	0.02	0.01	−0.04	0.02	0.03
Weekly health and contextual variables												
Pain interfering life				−1.65	1.30	0.21				−0.01	0.003	0.03
Low mood				−5.03	2.65	0.06				−0.002	0.01	0.81
Hours out-of-home				−3.34	0.87	<0.01				−0.01	0.003	0.02
Hours of daylight				−0.94	0.31	<0.01				< -0.01	0.001	0.66
Demographic variables												
Age in years				−0.42	0.56	0.45				0.003	0.001	<0.01
Gender (male)				−8.90	10.06	0.38				0.03	0.02	0.10
Years of education				−2.56	1.83	0.16				0.001	0.003	0.62
Race (White)				−10.72	14.21	0.45				0.05	0.02	0.01
Number of rooms in the house				−6.64	1.94	<0.01				0.003	0.004	0.37

### GEE Model With the Outcome Being Mobility Stability

**Figure 3:**
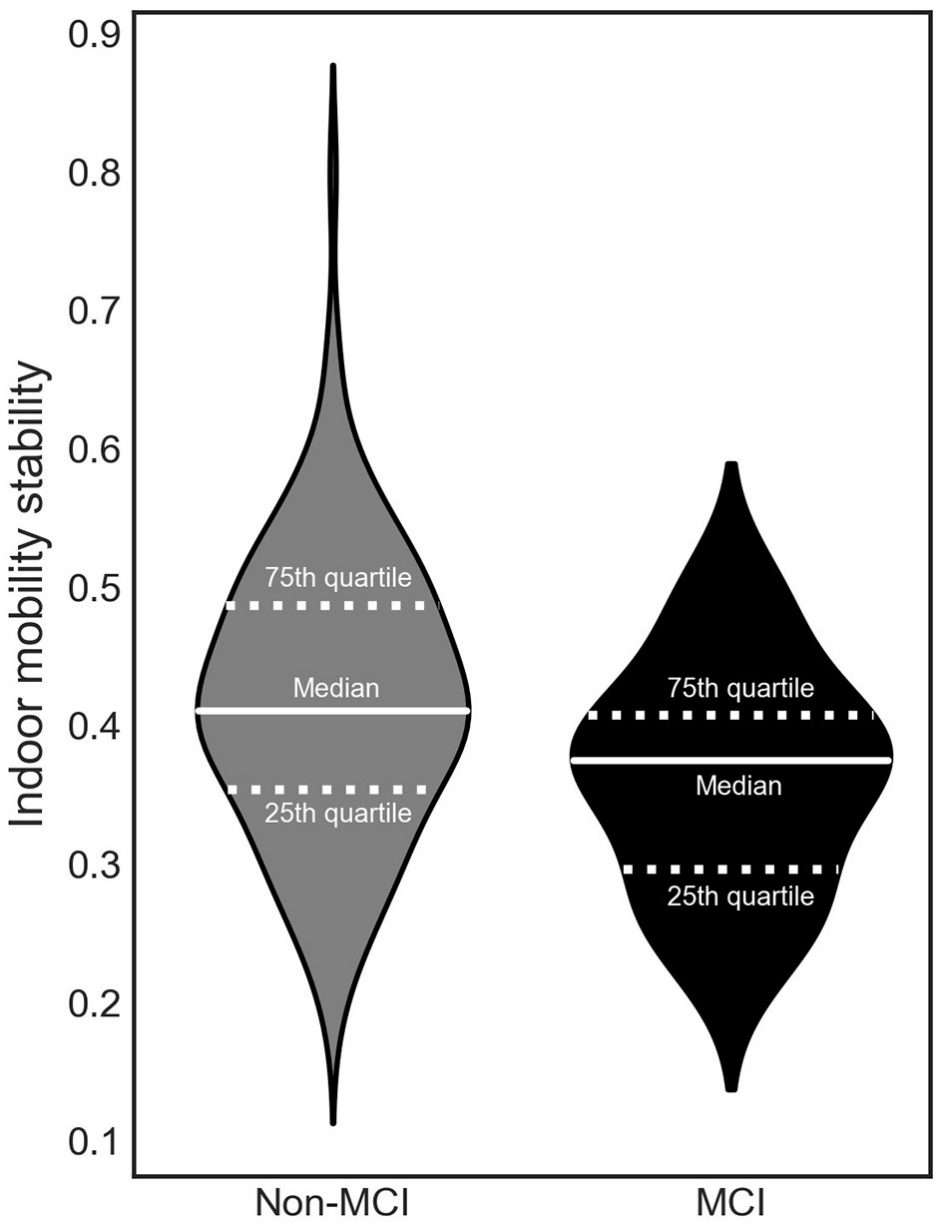
The relationship between indoor mobility stability and mild cognitive impairment (MCI).

A GEE model revealed that lower indoor mobility stability, meaning higher variability from day to day within a week, was associated with MCI status (β = −0.05, *p* = 0.01) (Figure 3). Results remained statistically significant after adjusting for age, gender, race, education, pain, low mood, number of rooms, hours of daylight, and time out-of-home (β = −0.04, *p* = 0.03) (Table 2).

## Discussion

In this study, we measured mobility at home using the number of transitions made across indoor geographic locations. We examined the psychometric properties of indoor mobility frequency and indoor mobility stability and associated them with physical and cognitive measures. We found that indoor mobility frequency and stability were reliable, valid, and clinically meaningful features. Participants with a faster gait speed, on average, exhibited a higher mobility frequency in the home compared with participants with a slower gait speed. Participants with MCI, on average, showed lower mobility stability in the home compared with the non-MCI group, and the group difference in the mobility stability yielded a large effect size. Considering the tremendous amount of time older adults spend inside their residences, monitoring indoor mobility using a pervasive-sensing system provides an ecologically valid extended window to delineate cognitive and functional status in older adults who live alone.

We established MCIDs of indoor mobility frequency and stability to define the smallest change to be meaningful to older adults. This analysis followed the 2018 revised Food Drug Administration (FDA) guidance for early Alzheimer's disease (37), suggesting a validated measurement should report a defined magnitude or threshold for treatment effects. The MCIDs we reported are useful to gauge how much change is needed to claim treatment efficacy in Alzheimer's disease and related disorders trials and how much change is needed within individuals who may be expected to claim clinical meaningfulness. Notably, there are many ways to measure MCIDs, including distribution-based methods [percentage-based personal thresholds, e.g., below 50th percentile of baseline (38)] or global rating of change scales (subjective reports of how much change do participants perceive) (39). The value of MCID can vary among subgroups (40). Although our study participants were comprised of those with and without MCI and included 22% who were African American, we did not have further diversity that would allow us to explore how MCIDs vary across other cognitive states or racial/ethnic groups more broadly.

Our results were similar to previous studies (14, 20, 22). Hashidate et al. developed a self-reported questionnaire to document how often individuals moved across rooms in the past week in 20 community-dwelling older adults (14). They found the number of transitions from the bedroom to other rooms was moderately correlated with the Functional Independence Measure (FIM) and timed up and go test (TUG). Austin et al. also found indoor mobility, measured by PIR motion sensors, increased with increasing walking speed (22). The relationships between the number of room-to-room transitions and gait speed may best be understood if room transitions are viewed as a general surrogate of the ability to complete activities of daily living (ADL). Many studies have shown that gait speed is a strong predictor of completing indoor ADLs such as preparing meals, taking medication, or doing household chores (41). Reduced indoor mobility frequency may indicate weakness, fatigue, depression, apathy, or decreased balance to navigate rooms to complete ADLs. In Schutz and colleagues' case reports (16), activity heat maps suggested 4–8 room-to-room transitions per h in their healthy 89-year-old with a MoCA score of 22. The authors also found a strong relationship between MoCA scores and coefficient of variation (CoV) of room transitions, which paralleled our finding.

Our study demonstrated that indoor mobility stability calculated by a non-parametric variable of 24-h rhythm was different between older adults with and without MCI. A previous study found that a 0.13–0.14 difference in the stability of activity/rest cycles (Cohen's *d* = 0.8–0.9) may distinguish moderate dementia from a normal control group (42). Our data suggested that a 0.1 difference in the indoor mobility stability (Cohen's *d* = 1) could distinguish MCI (earlier in the disease course) from a normal cognition group. It suggested our measure was more effective in detecting cognitive impairment. Several studies also found that a higher MMSE score and older age were associated with a greater interdaily stability of the sleep-wake cycle (43, 44). The linkage between cognition and indoor mobility stability in late life may result from several factors, such as biological clocks (45) or endocrine changes (46). For example, fluctuations in total sleep time (47), sleep efficiency, melatonin secretion or hyperglycemia (48, 49), and dietary preferences (50) were found in older adults with cognitive impairment. These fluctuations could lead to shifted daily patterns of routines. Therefore, lower indoor mobility stability observed in the MCI group could signify physiological impairment in the regulation of everyday routines. While our study did not collect information about biological timing systems or endocrine axes (e.g., melatonin, cortisol), this could be explored in future studies. The linkage between indoor mobility stability and sleep-wake circadian rhythms also warrants further research to support this hypothesis.

Some covariates showed statistical significance in the models. These results showed the importance of collecting multi-domain data to delineate indoor mobility. For example, demographics (age, race) were associated with indoor mobility stability. Studies have shown an age-related tendency for spending more time inside the home (4, 51). This may explain less time out-of-home and higher indoor mobility stability observed in those at older ages. Participants of different races and ethnicity varied in their indoor mobility stability. Reasons for this finding are unknown, but should be explored in future studies with larger sample sizes and similar proportions of races across different geographic regions to adjust for weather and rurality. Furthermore, weekly health such as the oscillation of pain could influence indoor mobility stability. It is possible that participants had good and bad days; when pain is low, they might be able to transit in and out of the home, while they might struggle with activity restrictions on other days. Further, contextual variables such as hours of daylight could influence a person's indoor mobility frequency. Previous studies showed that a higher temperature and certain seasons (winter) were associated with less likelihood of moving around at home (22, 52). Altogether, without collecting data from various modalities (surveys, sensors, in-person visits), we would not have been able to examine the interplay between indoor mobility, cognition, and functional status while controlling for confounding factors.

There are limitations to the current study. We quantified indoor mobility using five common indoor geographic home locations (primary bathroom, bedroom, kitchen, living room, entrance). Although this approach is helpful to standardize mobility across different sizes of residences, selected rooms may not cover all the indoor behaviors of an individual. We did not examine weekdays and weekends separately, yet a previous study has shown that the regularity of human behaviors was similar during weekdays and weekends (1). We also did not have the ability to examine temperature and seasonality in this cohort. The analytical approach used in the current study produced population average estimates instead of individual estimates. Although the MCI group on average exhibited lower indoor mobility stability, indoor mobility stability indeed varied across weeks within individuals. Since habitual home-mobility patterns have been shown to be highly individualized (22), future studies using an individual-specific trajectory approach are warranted to detect indoor mobility changes associated with MCI and dementia.

Sensor-based technologies have exploded in recent years, allowing remote, continuous measurement of older adults' mobility at home. Measuring mobility requires considering the context where the person is behaving. Our study used a ubiquitous, continuous sensing approach to monitor mobility in the home with the advantages of passive unobtrusive sensors and thus less burden on participants. We were able to conceptualize ecologically valid indoor mobility features. These mobility features showed reliability, validity, and clinical meaningfulness and further proved that a pervasive-sensing system deployed in homes could be useful in revealing cognitive and functional status for older adults who live alone.

## Conclusions

Older adults spend a considerable amount of time inside their residences; however, most research investigates out-of-home mobility and its health correlates. We measured indoor mobility using room-to-room transitions, tested their psychometric properties, and correlated indoor mobility with cognitive and functional status. We found that a higher indoor mobility frequency was associated with faster gait speed. We also found that a decrease in mobility stability was associated with MCI. These results demonstrate that mobility frequency and stability in the home are clinically meaningful and reliable features. Pervasive-sensing systems deployed in homes can objectively reveal cognitive and functional status in older adults who live alone.

## Data Availability Statement

The original contributions presented in the study are included in the article/supplementary files, further inquiries can be directed to the corresponding author.

## Ethics Statement

The studies involving human participants were reviewed and approved by the Institutional Review Boards of Oregon Health & Science University (IRB #2765, #17123, #4089) and Rush University (IRB # L03030302). The patients/participants provided their written informed consent to participate in this study.

## Author Contributions

C-YW planned the study, performed all statistical analyses and wrote the paper. HD, JK, and ZB supervised the data analysis and contributed to revising the paper. SG and NM extracted the data and contributed to revising the paper. LB, LS, CR, and ML contributed to revising the paper. All authors contributed to the article and approved the submitted version.

## Funding

This work received funding from the National Institute on Aging: P30AG024978, R01AG024059, P30-AG008017, and U2C AG054397 to JK; RF1AG22018, P3010161, and R01AG17917 to LB; P30AG066518-01 Development Program to C-YW; P30AG024978-15 Roybal Pilot and P30AG066518-02 Development Program to ML; the Department of Veterans Affairs Health Services Research and Development: IIR 17-144; the National Center for Advancing Translational Sciences: UL1 TR002369, and the Pacific Northwest National Laboratory: Hartford Gerontological Center Interprofessional Award to ML.

## Conflict of Interest

JK has received research support awarded to his institution (Oregon Health & Science University) from the NIH, NSF, the Department of Veterans Affairs, USC Alzheimer's Therapeutic Research Institute, Merck, AbbVie, Eisai, Green Valley Pharmaceuticals, and Alector. He holds stock in Life Analytics Inc. for which no payments have been made to him or his institution. ZB hold stock in Life Analytics Inc. for which no payments have been made to him or his institution. The remaining authors declare that the research was conducted in the absence of any commercial or financial relationships that could be construed as a potential conflict of interest.

## Publisher's Note

All claims expressed in this article are solely those of the authors and do not necessarily represent those of their affiliated organizations, or those of the publisher, the editors and the reviewers. Any product that may be evaluated in this article, or claim that may be made by its manufacturer, is not guaranteed or endorsed by the publisher.
